# Elimination of stem-like cancer cell side-population by auranofin through modulation of ROS and glycolysis

**DOI:** 10.1038/s41419-017-0159-4

**Published:** 2018-01-24

**Authors:** Guo-Xin Hou, Pan-Pan Liu, Shengyi Zhang, Mengqi Yang, Jianwei Liao, Jing Yang, Yumin Hu, Wen-Qi Jiang, Shijun Wen, Peng Huang

**Affiliations:** 1 Sun Yat-Sen University Cancer Center, State Key Laboratory of Oncology in South China, Collaborative Innovation Center for Cancer Medicine, Guangzhou, 510060 Guangdong China; 2 Department of Medical Oncology, Sun Yat-sen University Cancer Center, Guangzhou, 510060 Guangdong China; 3 School of Pharmaceutical Sciences, Sun Yat-sen University, 132 Wai huan East Road, Guangzhou, 510006 Guangdong China; 4 Department of Translational Molecular Pathology, Unit 951, The University of Texas MD Anderson Cancer Center, 1515 Holcombe Boulevard, Houston, TX 77030 USA

## Abstract

Cancer side-population (SP) represents a sub-population of stem-like cancer cells that have an important role in drug resistance due to their high expression of the ATP-binding cassette transporter ABCG2 involved in drug export. Auranofin (AF), a clinical drug of gold complex that is used in treatment of rheumatoid arthritis, has been reported inducing tumor antiproliferation. However, whether AF can impact SP cells remains unclear. Our study showed that AF caused a depletion of SP cells and a downregulation of stem cell markers, and impaired their ability to form tumor colonies in vitro and incidence to develop tumors in vivo of lung cancer cells. Reactive oxygen species (ROS) had an important role in mediating AF-induced depletion of SP cells, which could be reversed by antioxidant NAC. Further study revealed that AF could also cause ATP depletion by inhibition of glycolysis. The depletion of cellular ATP might impair the function of ABCG2 pump, leading to increased drug accumulation within the cells and thus enhancing anticancer activity of chemotherapeutic agents such as adriamycin. Synergistic effect of AF and adriamycin was demonstrated both in vitro and in vivo. Simultaneous increase of ROS and inhibition of glycolysis is a novel strategy to eliminate stem-like cancer cells. Combination of AF with adriamycin seems to be promising to enhance therapeutic effectiveness.

## Introduction

Cancer stem cells (CSCs) are a small sub-population of cells within a tumor that possess the capacity to self-renew and generate downstream lineages of cancer cells comprising the tumor bulks^1^. CSCs are considered as the root of tumor initiation and have an important role in drug resistance and tumor recurrence, thus targeting the CSCs has great therapeutic potential^2–4^. An important property of CSCs is their high expression of ATP-binding cassette transporter proteins, especially ABCG2 which actively efflux many chemotherapeutic drugs including adramycin (ADM) and taxol. Owing to the existence of ABCG2, a DNA binding dye, Hoechst 33342 can be pumped out as a substrate, serving as the basis of side-population (SP) assay to identify the stem-like cancer cells in certain types of cancers^5,6^. The existence of CSCs is currently regarded as a major challenge in cancer treatment. Therefore, it is extremely important to develop effective strategies to eliminate CSCs using proper therapeutic agents. Recent studies showed that potential strategies against CSCs included inhibition of the survival signaling pathways relevant to CSCs, blockage of the stromal microenvironment protection for CSCs, and targeting the specific metabolic alterations in CSCs^7–9^.

Previous study showed that SP cells exhibited increased glycolytic activity compared with the non-SP cells^10,11^. Inhibition of glycolysis using compounds such as 3-bromo-2-oxopropionate-1-propyl ester (3-BrOP) could effectively decrease the proportion of SP cells in vitro and impair their tumorigenicity in vivo^10^, suggesting that glycolytic pathway might be a potential target for eradicating CSCs. Yuan et al.^11^ reported that 3-BrOP was able to inhibit two glycolytic enzymes, glyceraldehyde-3-phosphate dehydrogenase (GAPDH) and hexokinaseII (HKII), and preferentially killed glioblastoma stem cells (GSCs) that have high glycolytic activity. Recent studies showed that mitochondria could also be a potential therapeutic target to kill tumor-initiating cells (TICs)^12,13^.

In addition to the significant impact of glycolytic metabolism on cell stemness, reactive oxygen species (ROS) are also known to have an important role in promoting cell differentiation/senescence and affecting stem cells^14,15^. Thus, it might be effective to eliminate cancer stem cells by simultaneously increasing ROS generation and inhibiting glycolysis. Auranofin (AF), a clinical drug of gold complex, is used in treatment of rheumatoid arthritis^16^. It has been reported inducing tumor antiproliferation and apoptosis in various types of tumor by inhibiting the function of thioredoxin reductase (TrxR) and 19S proteasome-associated deubiquitinases ^17–19^. However, whether it can impact the SP cells remains unclear. Interestingly, we indeed found that AF could effectively deplete SP cells through increasing ROS generation and inhibition of the glycolytic enzyme hexokinase. Moreover, synergistic effect of AF and adriamycin (ADM) was demonstrated both in vitro and in vivo, indicating that a combination of AF with conventional chemotherapeutic agents may be a promising novel strategy to treat tumors.

## Results

### Depletion of stem-like SP cells by auranofin

**Fig. 1:**
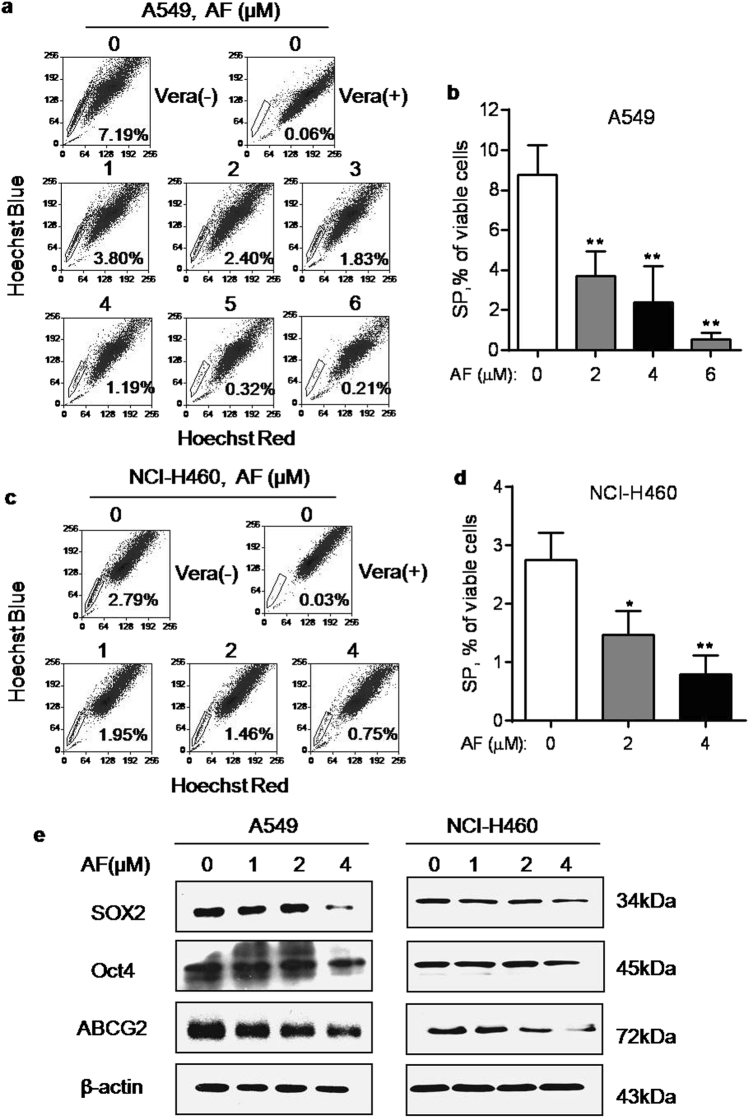
Effect of AF on SP cells and the expression of stem cell markers **a** A549 cells were treated with various concentrations of AF as indicated for 24 h, cells stained with Hoechst 33342 for 90 min and then analyzed for SP cells by flow cytometry. SP cells were gated as the sub-population that disappeared after treatment with the ABC pump inhibitor verapamil (Vera). **b** Quantitative data of three separate experiments described in **a**. Each bar represents mean ± SD, **p < 0.01. **c** NCI-H460 cells were incubated with various concentrations of auranofin as indicated for 24 h, cells stained with Hoechst 33342 for 90 min and then analyzed for SP cells by flow cytometry. **d** Quantitative data of three separate experiments described in **c**. Each bar represents mean ± SD, *p < 0.05; **p < 0.01. **e** A549 cells and NCI-H460 cells were incubated with the indicated concentrations of auranofin for 24 h, and the expression of Sox2, Oct4, and ABCG2 was then measured by western blot analysis. β-actin was blotted as protein loading control

Four human lung cancer cell lines A549, NCI-H460, Sk-MES-1, and Hcc827 cells were tested for their SP percentage. The only non-small cell lung cancer cell lines A549 and NCI-H460 were found to contain considerable portion of SP, consistent to the previous report^10^ and however, the SP cells in the other two cell lines Sk-MES-1 and Hcc827 were scarce (Supplementary Fig. 1). Therefore, only A549 and NCI-H460 were used to test the cytotoxic effect of AF on the overall cell survival and the specific impact on SP cells. A549 and NCI-H460 cells were treated with various concentrations of AF for 72 h, and cell viability was determined by  3-(4,5-dimethylthiazol-2-yl)-5-(3-carboxymethoxyphenyl)-2-(4-sulfophenyl)-2H-tetrazolium (MTS) assay. AF decreased the cell viability of A549 cells in concentration-dependent manner with an IC_50_ value of 4 μM. The IC_50_ value for NCI-H460 cells was 2 μM (Supplementary Fig. 2). We then analyzed the impact of AF on SP cells. After A549 cells were exposed to various concentrations of AF for 24 h, the attached viable cells were collected for SP analysis. As shown in Fig. 1a and b, the population of SP cells was significantly decreased in a concentration-dependent manner after AF treatment. At the IC_50_ concentration (4 μM), AF induced a depletion of SP cells to 1.2%. These data suggested that AF was able to preferentially eliminate stem-like SP from the whole cancer cell population. Similar impact of AF on SP cells was also observed in NCI-H460 cells (Fig. 1c, d). It is worth noting that a high dose of AF at 10 μM was needed to induce apoptosis (data not shown). Consistent with the decrease in SP cells, SOX2, Oct4, and ABCG2, that are markers of stem cells^20–22^, were also found decreased after AF treatment in A549 and NCI-H460 cells (Fig. 1e).

Cancer cells are notorious for their plasticity. Cancer stem cells can differentiate and generate non cancer stem cells. In turn, non-CSCs can become cancer stem cells by de-differentiation. To test the impact of AF on SP cells and plasticity, we first incubated A549 cells with 4 μM AF for 24 h to deplete SP cells, and then switched the cells to fresh medium without AF. The percentage of SP cells decreased from 7.54 to 2.08% when A549 cells were treated by AF. However, the subsequent removal of AF from the culture medium led to a time-dependent increase of SP cells (Supplementary Fig. 3a). This unique phenomenon was consistently observed in three separate experiments (Supplementary Fig. 3b), suggesting that although AF was able to effectively deplete SP cells, the remaining cancer cells might be able to regenerate SP cells after removal of the drug.

### Impact of AF on cancer colony/sphere formation in vitro and tumor development in vivo

**Fig. 2:**
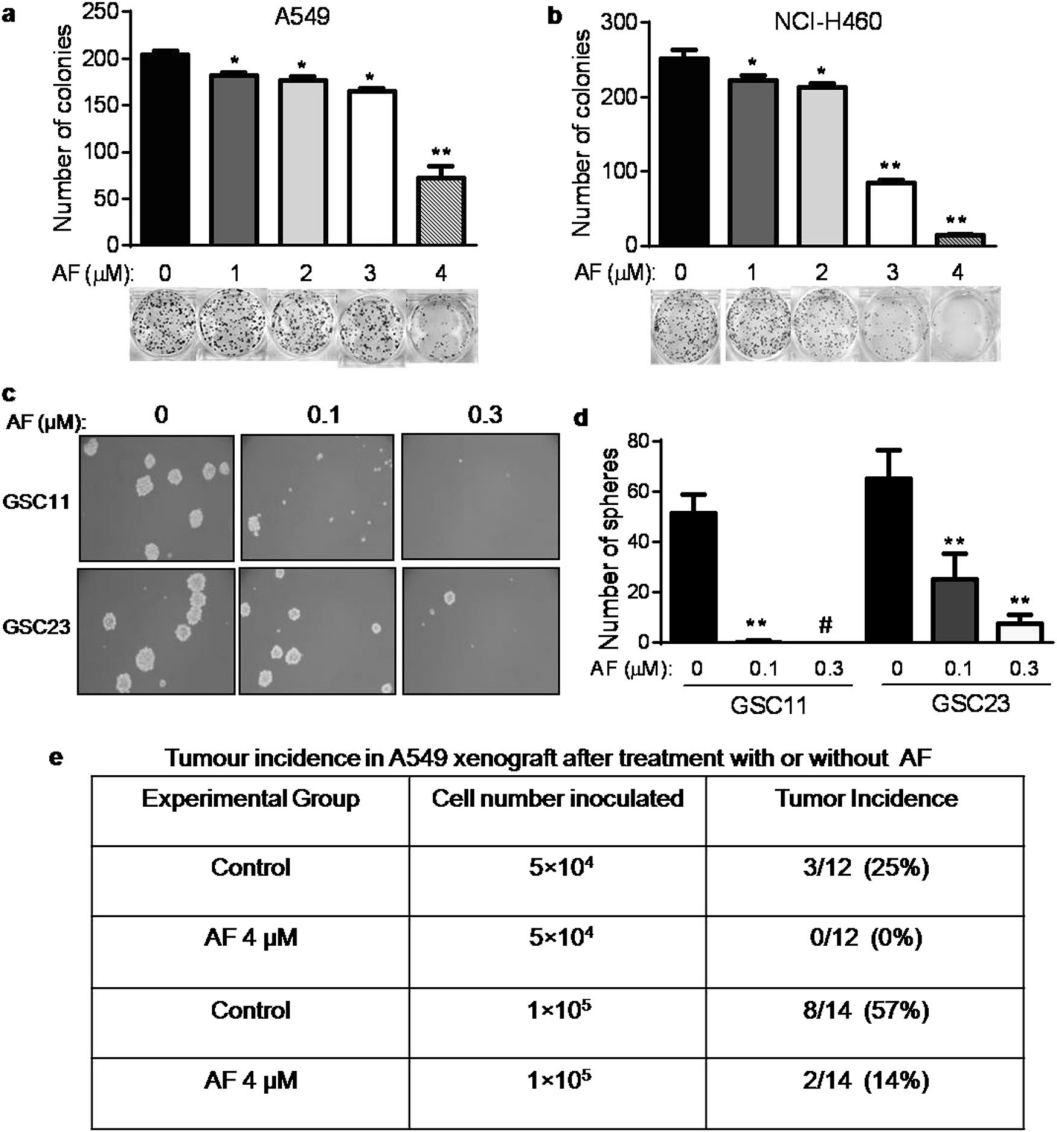
Impact of AF on cancer colony/sphere formation in vitro and tumor development in vivo **a**, **b** A549 and NCI-H460 cells were incubated with the indicated concentrations of AF. After 14 days, cell colonies were fixed, stained, and counted. Each bar represents mean ± SD, *n* = 3 separate experiments; *p < 0.05; **p < 0.01. **c**, **d** GSC11 and GSC23 cells were plated in six-well plates at a density of 800 cells per well, and treated with the indicated concentrations of AF. Tumor spheres were counted on day 12. Each bar represents mean ± SD, *n* = 3 separate experiments; **p < 0.01; ^#^no sphere formed. **e** A549 cells were first treated with 4 μM AF for 24 h, and then the viable cells were inoculated subcutaneously into the flanks of the mice at 5 × 10^4^ or 1 × 10^5^ cells per injection site. The mice were observed for tumor development without further drug treatment

As the ability to form colonies and tumor spheres is an important feature of cancer stem cells, we first tested the effect of AF on colony formation in A549 and NCI-H460 cells. Exposure of the cancer cells to 4 μM AF significantly reduced colony formation (Fig. 2a, b), indicating that the in vitro tumorigenic capacity of the cancer cells were impaired by AF. GSC11 and GSC23 originally derived from glioblastoma multiforme (GBM) surgical specimens exhibit the stem cell characteristics of self-renewal and the capacity of differentiation^23^. Thus, we tested the effect of AF on GSC11 and GSC23. The compound decreased cell viability and sphere formation of these two glioblastoma stem cell lines in a concentration-dependent manner (Fig. 2c, d; Supplementary Fig. 4). Our data collectively indicated that AF could effectively impact cancer cell stemness in both lung cancer cells and glioblastoma stem cells.

As the tumorigenic potential in vivo is a critical indicator of stemness, we further tested the effect of AF on tumorigenicity. A549 cells were treated with AF (4 µM) for 24 h in vitro, harvested, washed, and re-suspended in serum-free medium (RPMI1640), and subsequently inoculated subcutaneously into the flanks of athymic nude mice, with 5 × 10^4^ or 1 × 10^5^cells per injection site. The mice were then monitored for tumor formation without further drug treatment. As shown in Fig. 2e, pre-treatment of A549 cells with AF substantially reduced the incidence of tumor formation, implying that AF could impair tumorigenicity of cancer cells in vivo.

### Role of ROS in AF-induced depletion of SP cells

**Fig. 3:**
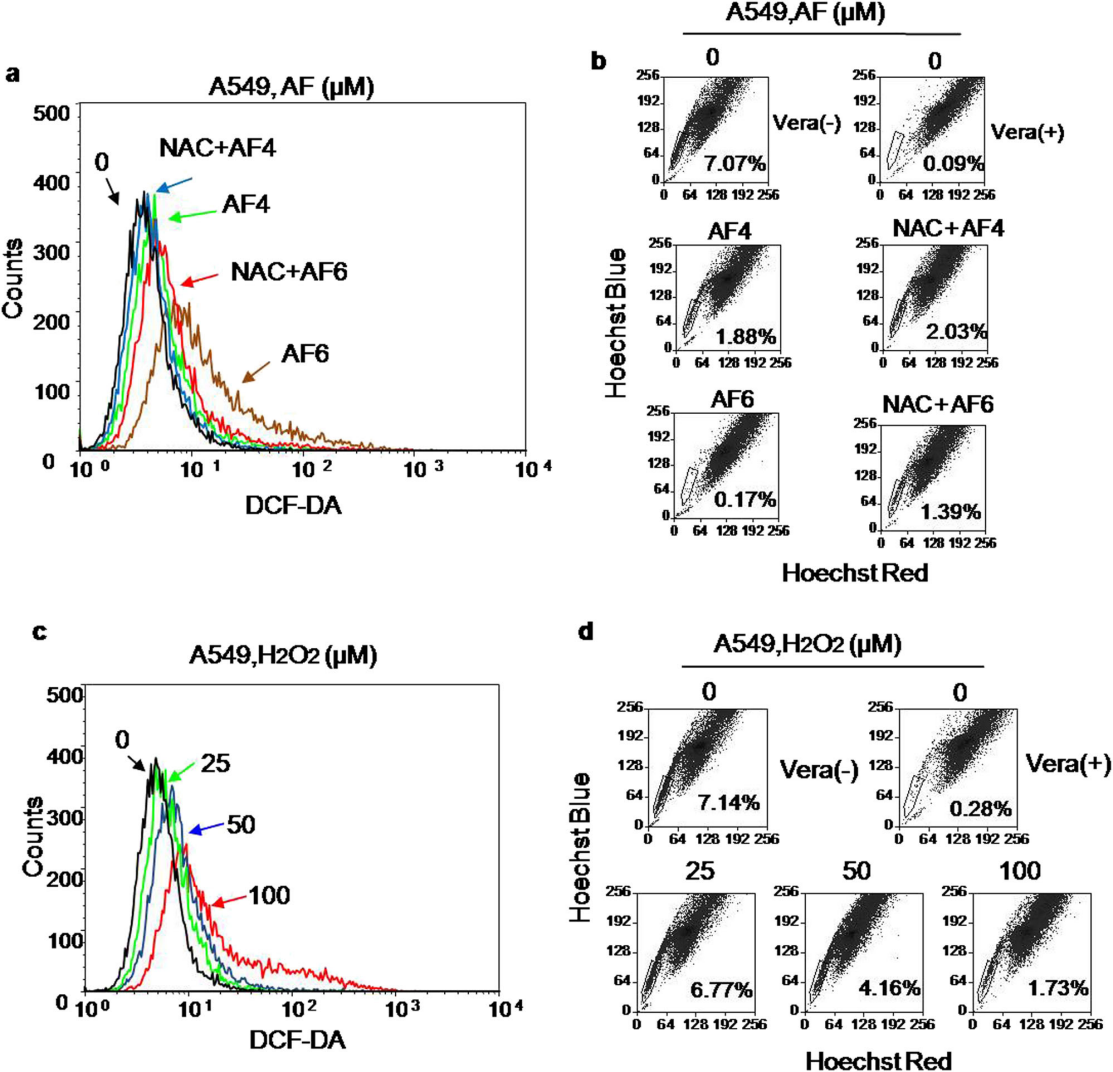
Role of ROS in mediating AF-induced depletion of SP cells **a** Induction of ROS increase by AF and its partial reversion by an antioxidant NAC. A549 cells were pre-treated with 3 mM NAC for 1 h, washed, and then incubation with auranofin (4–6 μM) for 24 h. ROS levels were measured by staining the cells with DCF-DA, followed by flow cytometry analysis. **b** AF-induced depletion of SP cells was partially prevented by pre-treatment with NAC. A549 cells were treated with 3 mM NAC for 1 h, washed, and then incubated with the indicated concentrations of auranofin for 24 h. SP cells were analyzed by flow cytometry. **c** A549 cells were treated with the indication concentrations of H_2_O_2_ for 24 h, and cellular ROS levels were measured by flow cytometry analysis after the cells were stained with DCF-DA. **d** A549 cells were treated with the indicated concentrations of H_2_O_2_ for 24 h, and SP cells was measured by flow cytometry

ROS have an important role in redox signaling and high concentrations of ROS promote cell differentiation and senescence^14,15^. As AF is a potent inhibitor of TrxR that affects cellular redox status, we postulated that this compound might impact SP cells via a ROS-mediated mechanism. To test this possibility, we first measured cellular ROS before and after AF treatment. AF caused a concentration-dependent increase of cellular ROS, which could be partially neutralized by pre-incubation with an antioxidant N-acetyl-l-cysteine (NAC), a widely employed reducing agent to study ROS-mediated cellular events (Fig. 3a; Supplementary Fig. 5a). Consistently, NAC was able to partially reverse the impact of AF on SP cells (Fig. 3b; Supplementary Fig. 5b), suggesting that ROS might at least in part mediate the effect of AF on SP cells. To directly test the role of ROS, we treated A549 cells with various concentrations of H_2_O_2_, and measured the change in SP cells. Incubation of the cells with H_2_O_2_ caused a concentration-dependent increase in cellular ROS and a proportional decrease in SP cells (Fig. 3c, d; Supplementary Fig. 5c, d). These data together suggest that ROS might have a role in affecting stemness of cancer cells treated by AF. It is known that AF inhibits TrxR, which is an important antioxidant protein. Thus, we tested whether overexpression of TrxR1 could reverse the AF-induced ROS increase. As shown in Supplementary Fig. 5e–f, overexpression of TrxR1 by infection of A549 cells with lentiviral vector containing TrxR1 construct was able to partially reverse the effect of AF on ROS, whereas the empty control vector exhibited no significant effect.

### Induction of ATP depletion by AF and its impact on hexokinase activity

**Fig. 4:**
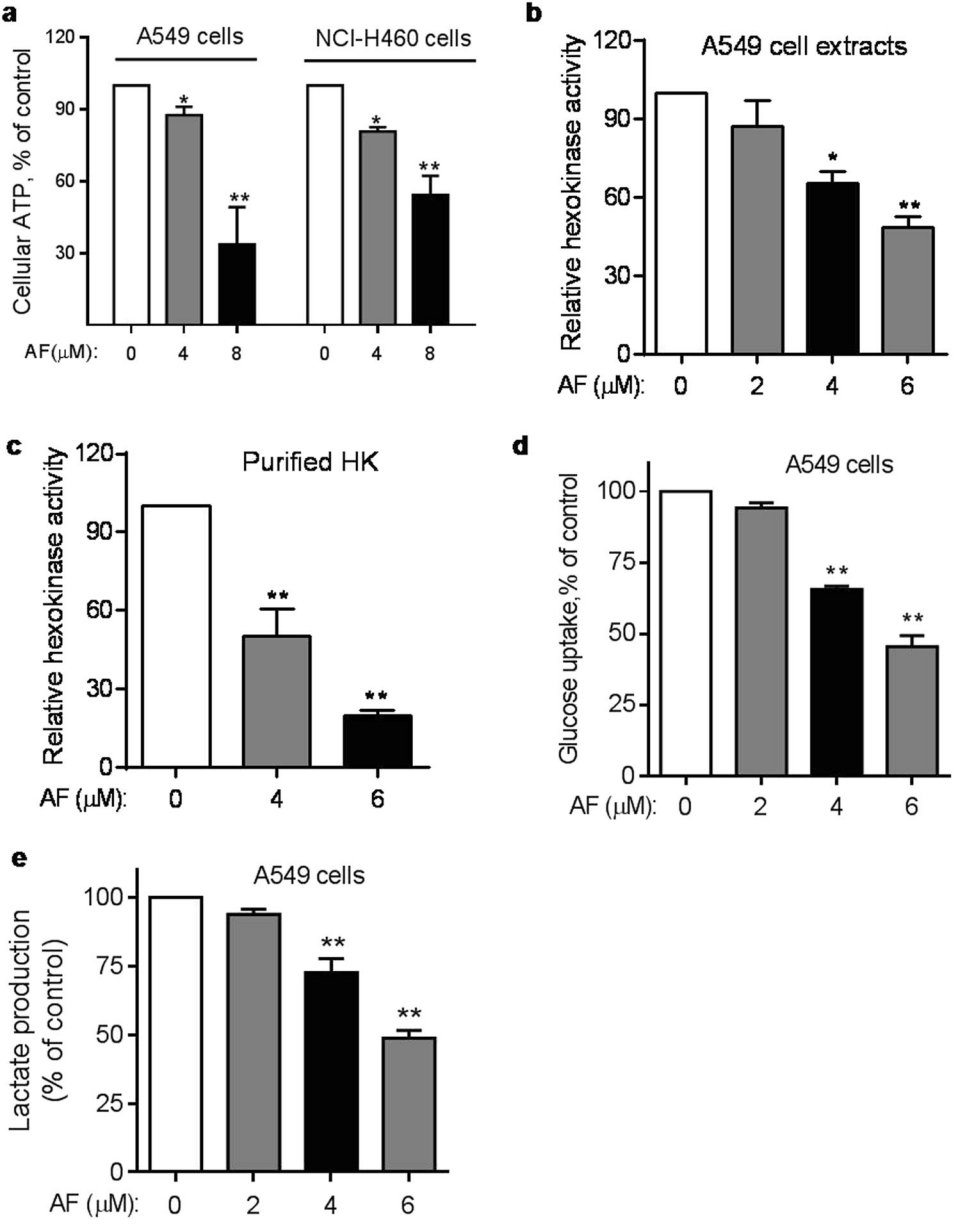
Depletion of cellular ATP and inhibition of glycolysis by AF **a** A549 and NCI-H460 cells were incubated with the indicated concentrations of AF for 6 h, and intracellular ATP levels were measured. **b** A549 cells were incubated with the indicated concentrations of AF for 6 h. Cellular proteins were then extracted and used for assay of hexokinase enzyme activity. **c** Purified hexokinase enzyme was pre-incubated with various concentrations of AF for 10 min, and the enzyme activity was then measured. **d**, **e** Inhibition of glucose uptake and lactate production by AF. A549 cells were treated with the indicated concentrations of AF in fresh medium for 12 h. The culture media from each sample was then collected for analysis of glucose and lactate. Each bar represents mean ± SD of three separate measurements; *p < 0.05; **p < 0.01

Considering that ABCG2 is a major ABC transporter in cancer stem cells and its pump function is dependent on ATP^5,6^, we tested if AF could affect cellular ATP and thus impact the function of ABCG2 transporter. We first measured intracellular ATP levels after A549 or NCI-H460 cells were treated with AF. ATP levels in both cell lines were substantially decreased after the cells were incubated with AF for as early as 6 h (Fig. 4a). Further study showed that AF was able to inhibit hexokinase (HK), a key enzyme in the glycolytic pathway that catalyzes the first step of glycolysis. Incubation of A549 cells with various concentrations of AF led to a dose-dependent inhibition of cellular HK activity. We noted that 2 μM AF did not significantly inhibit HK, whereas substantial inhibition was observed at higher drug concentrations (4–6 μM) (Fig. 4b). The ability of AF at 4–6 μM to inhibit HK was further demonstrated using purified hexokinase (Fig. 4c). Consistently, cellular glucose uptake and lactate production were also significantly suppressed by AF at 4–6 μM (Fig. 4d, e). These data together suggest that AF might cause ATP depletion through inhibition of HK to suppress glycolysis.

**Fig. 5:**
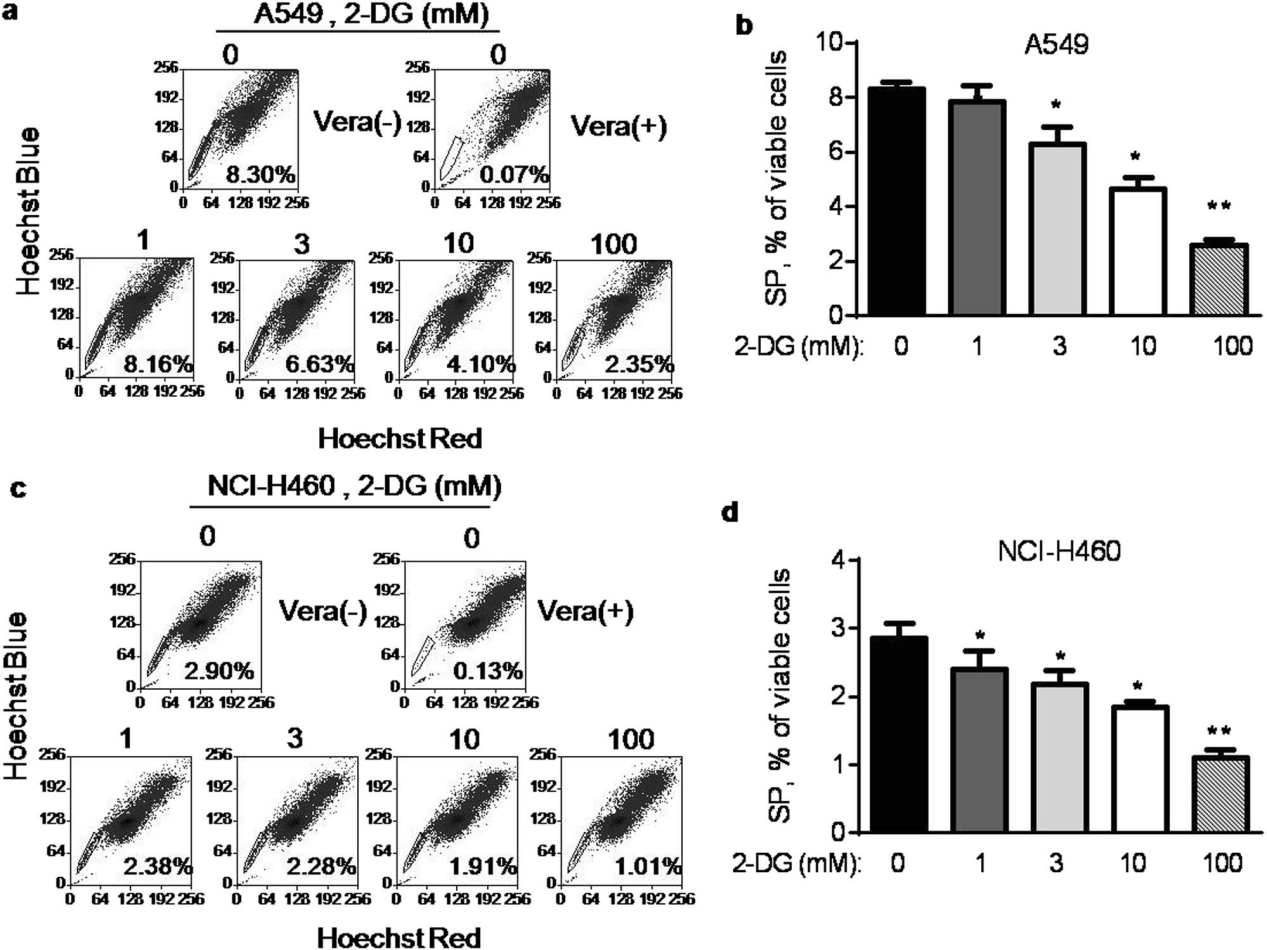
Effect of 2-deoxyglucose on SP cells in vitro **a** A549 cells were treated with the indicated concentrations of 2-deoxyglucose (2-DG) for 24 h, stained with Hoechst 33342 for 90 min, and then analyzed for SP by flow cytometry. **b** Quantitative data of three separate experiments described in **a**. Each bar represents mean ± SD, *p < 0.05; **p < 0.01. **c** NCI-H460 cells were treated with the indicated concentrations of 2-DG for 24 h, stained with Hoechst 33342, and then analyzed for SP by flow cytometry. The number within each panel shows the percentage of SP cells. **d** Quantitative data of three separate experiments described in **c** Each bar represents mean ± SD of three separate measurements, *p < 0.05; **p < 0.01

To evaluate the impact of HK inhibition on SP cells, we tested if 2-deoxyglucose (2-DG), a known inhibitor of HK^24^, could cause any decrease in SP cells. 2-DG induced a decrease of SP cells in a dose-dependent manner in A549 and NCI-H460 cells. We noted that high concentrations of 2-DG were needed to induced depletion of SP cells (Fig. 5), while the glycolytical activity was substantially suppressed at high doses of 2-DG (Supplementary Fig. 6).

### Synergistic therapeutic activity of AF in combination with ADM

**Fig. 6:**
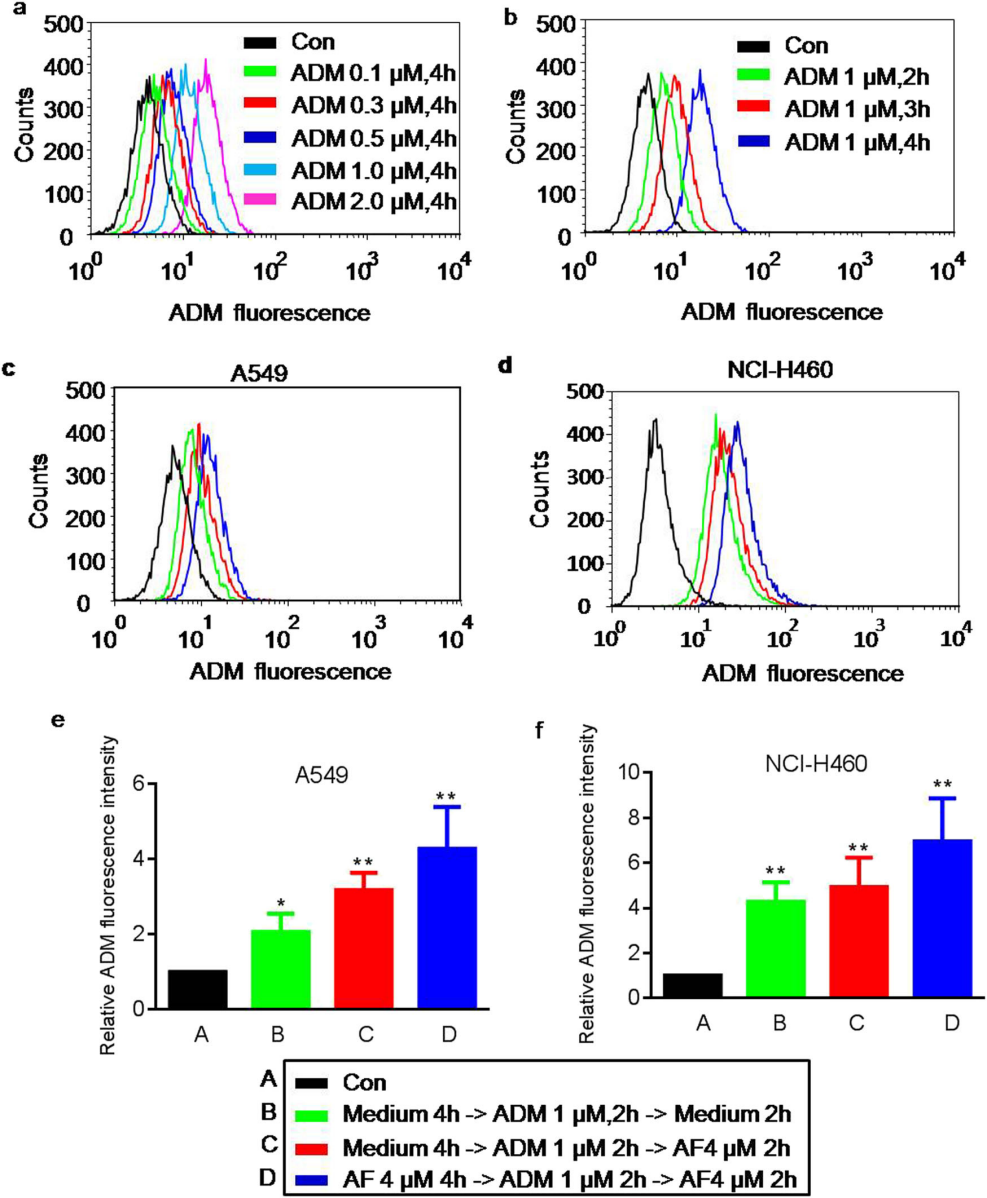
Synergistic activity of AF in combination with ADM against lung cancer cells in vitro **a**, **b** Cellular uptake of ADM measured by flow cytometry analysis of ADM fluorescence. A549 cells were treated with various concentrations of ADM for 4 h, and ADM fluorescence was measured by flow cytometry; cells were treated 1 µM ADM for various time points, and ADM fluorescence was measured by flow cytometry. **c**, **d** Effect of AF on cellular uptake of ADM. A549 cells or NCI-H460 cells were incubated with drugs under the four different conditions as indicated. The intensity of fluorescent signal of ADM in each sample was measured by flow cytometry. **e**, **f** Quantitative data of three separate experiments described in **c** and **d**. Each bar represents mean ± SD of three separate measurements, *p < 0.05; **p < 0.01

On the basis of the finding that AF was able to induce cellular ATP depletion (Fig. 4a), we postulated that the ATP depletion might compromise the function of the ATP-dependent ABCG2 to export chemotherapeutic drug ADM^25,26^ and thus reduce its anticancer activity. To test this possibility, we first used the fluorescent property of ADM to measure the cellular uptake of this compound, and then evaluated the impact of AF on intracellular content of ADM. The cellular uptake of ADM was concentration-dependent and time-dependent (Fig. 6a, b). A proper concentration of ADM (1 μM) and incubation time (2 h) as appropriate conditions were finally used to evaluate the impact of AF on cellular retention of ADM. As shown in Fig. 6c–f, A549 and NCI-H460 cells were divided into control group (without drug treatment) and three treatment groups. In treatment group 1 and 2, the cells were incubated in fresh medium for 4 h and then treated with 1 μM ADM for 2 h, washed, and finally incubated in fresh medium (treatment group 1) or in fresh medium containing 4 μM AF (treatment group 2) for another 2 h. In treatment group 3, cells were pre-incubated with 4 μM AF for 4 h and then treated with 1 μM ADM for 2 h, washed, and finally incubated in fresh medium containing 4 μM AF for another 2 h. Compared with the control group and treatment group 1, the intracellular ADM fluorescence was substantially higher in treatment groups 2 and 3, indicating that AF could increase intracellular ADM retention.

**Fig. 7:**
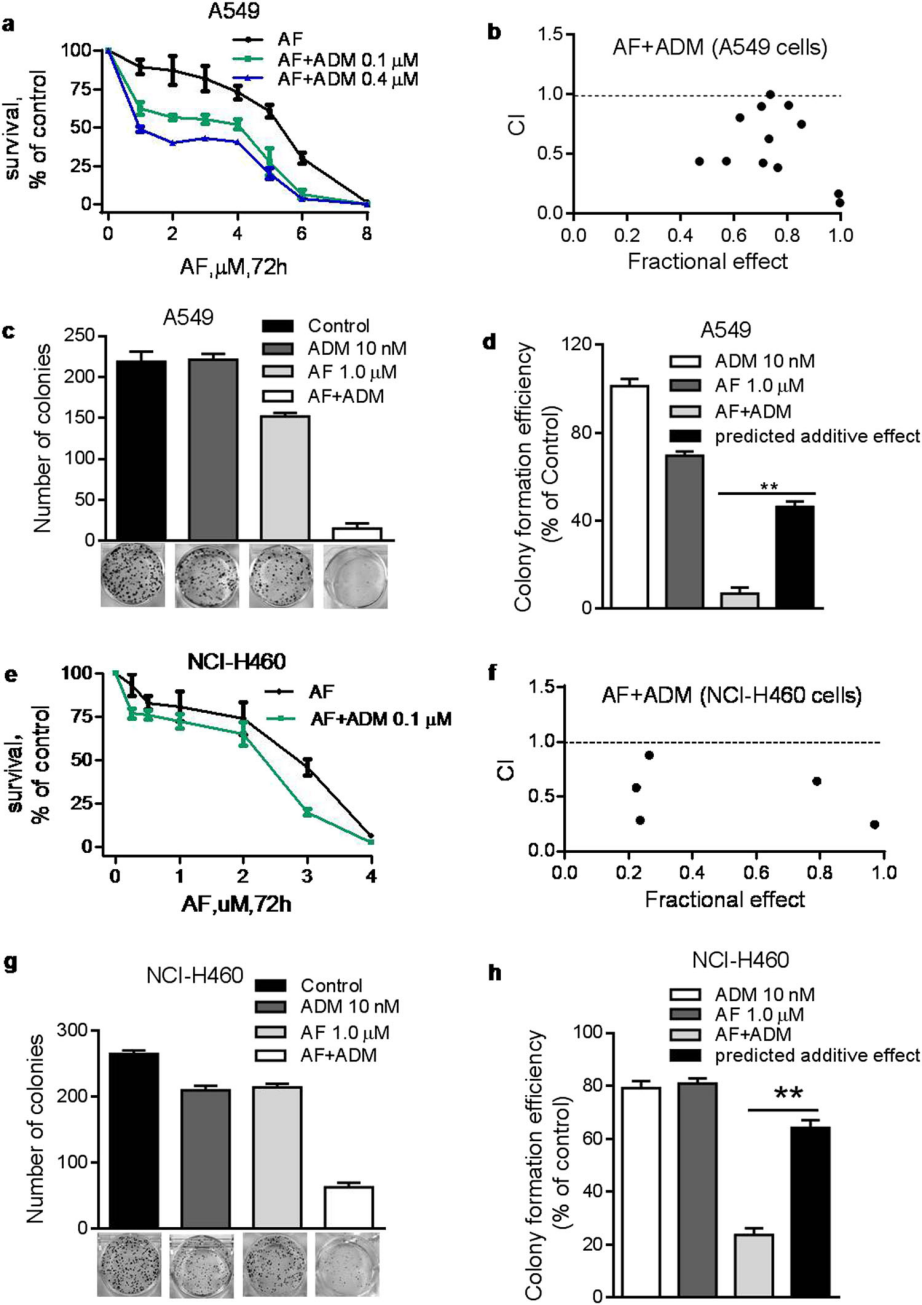
Synergistic activity of AF in combination with ADM against lung cancer cells in vitro **a**, **e** A549 and NCI-H460 cells were incubated with various concentrations of AF with or without different concentrations of ADM for 72 h. Cell viability was determined by MTS assay. **b**, **f** Analysis of drug CI of AF and ADM in A549 and NCI-H460 cells. CI values were calculated using the Calcusyn software (Biosoft). **c**, **g** A549 and NCI-H460 cells were incubated with AF, ADM, or their combination. The numbers of cell colonies were counted after 14 days. **d**, **h** Comparison of the observed drug combination (AF + ADM) effect (third column) and the predicted additive effect (fourth column) on colony formation. The predicted additive viable cell percentage was calculated by multiplying the respective % of viable cells treated by either AF or ADM alone. Each bar represents mean ± SD of three separate measurements, **p < 0.01

On the basis of the above observation, we then tested whether the combination of AF with ADM might have synergistic activity against A549 cells. MTS assay demonstrated that AF synergistically potentiated the cytotoxic effect of ADM in A549 cells, as evidenced by the combination index (CI) analysis, in which most of the CI values were <1.0 (Fig. 7a, b). The synergistic effect was further confirmed using colony formation assay (Fig. 7c, d) and apoptosis assay (Supplementary Fig. 7a, b). Similar synergistic effect between AF and ADM was also observed in another lung cancer cell line NCI-H460 (Fig. 7e–h; Supplementary Fig. 7c, d). We also tested the effect of AF and its combination with ADM on the expression of cancer stem cell markers including SOX2, Oct4, and ABCG2 (Supplementary Fig. 7e). AF (4 μM) caused a substantial decrease in expression of all three stem cell markers in A549 cells, whereas ADM did not induced such change. Combination of AF and ADM did not further reduce the stem cell marker expression compared to AF alone. These data are consistent with the conclusion that AF and ADM killed CSCs and non-CSCs, respectively.

### In vivo therapeutic activity of AF and ADM

**Fig. 8:**
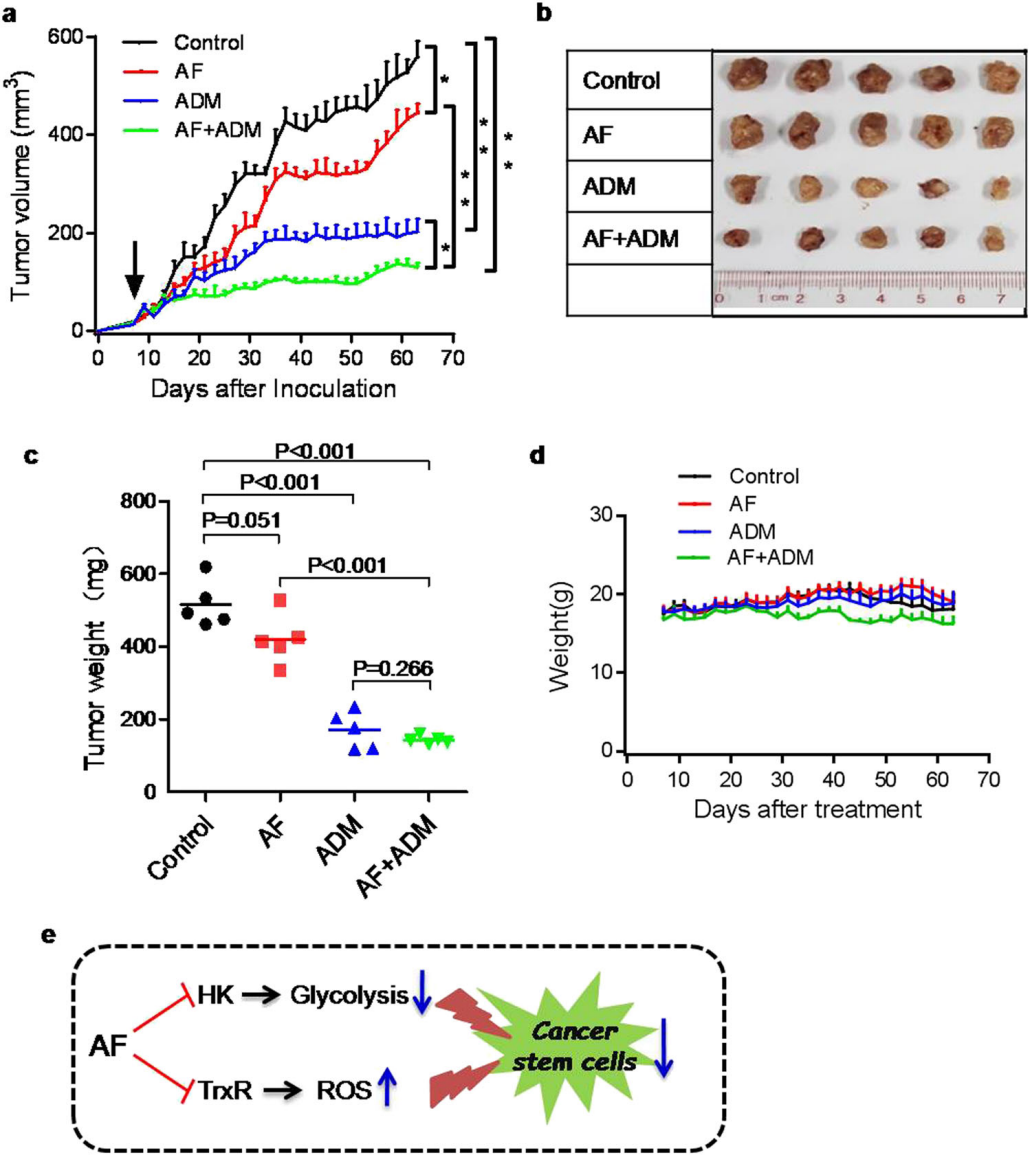
In vivo therapeutic effect of AF, ADM, and their combination in mice bearing A549 tumor xenografts **a** Mice were inoculated with A549 cells and treated with AF (10 mg/kg, i.p.), ADM (5 mg/kg, i.v.), or their combination. Tumor sizes were measured every other day using a caliper. *p < 0.05; **p < 0.01. **b**, **c** Comparison of tumor sizes and weights of different treatment groups at the end of animal experiment (9 weeks). *p* values were calculated using the two-tailed Student *t-*test. **d** Body weights of mice were measured every other day. Each time point represents mean ± SD of five mice in each group. **e** The proposed model of AF impacting on cancer cells

On the basis of the evidence that a combination of AF with ADM could synergistically kill cancer cells in vitro, we further tested if such synergistic effect could be achieved in vivo in tumor xenograft models. A549 cells were subcutaneously injected into the flanks of mice (1.5 million cells per injection site). When the tumors were palpable on day 7 after inoculation, the mice were randomly divided into four groups: (1) Control mice without drug treatment; (2) Mice treated with AF (10 mg/kg, i.p., five times per week for 6 weeks); (3) Mice treated with ADM (5 mg/kg, i.v., once per week for 6 weeks); (4) Mice treated with a combination of AF (10 mg/kg, i.p.) and ADM (5 mg/kg, i.v.). As shown in Fig. 8a–c, treatment with AF or ADM alone exhibited moderate inhibitory effect on tumor growth. Combination of both drugs resulted in a significant increase in therapeutic activity, as evident by a substantial delay in tumor growth. These drug treatment conditions seemed well tolerated by the animals as there was not significant loss in body weights (Fig. 8d).

## Discussion

As CSCs were first identified in acute myeloid leukemia, the existence of this rare sub-population of cancer cells have been found in various solid tumors including brain, breast, lung, and pancreas tumor^27–32^. CSCs present a major challenge in clinical cancer treatment due to their resistance to many chemotherapeutic agents. Development of effective therapeutic strategies to eradicate CSCs would greatly improve cancer treatment outcome and prolong the survival of cancer patients. Preferential elimination of CSCs based on their intrinsic metabolic alterations is an attractive therapeutic strategy. Our previous study showed that inhibition of glycolysis could effectively kill stem-like SP cells^10^. In addition to active glycolysis as a metabolic feature, CSCs seem to require a low cellular ROS content to maintain their stemness. A novel derivative of naturally occurring phenethyl isothiocyante was found to deplete CSCs by transiently increasing intracellular ROS in our other study^33^. As such, compounds that affect both glycolysis and ROS generation might be highly effective in elimination of CSCs.

In this study, we found that AF, an anti-rheumatic drug used in clinic, could effectively eradicate CSCs through redox modulation (increasing ROS) and inhibition of glycolysis (Fig. 8e). In addition, depletion of cellular ATP by AF could in turn compromise the drug efflux pump activity of ABCG2, leading to more intracellular retention of ADM in cancer cells. These findings may explain why the combination of AF with ADM exhibited potent synergistic effect both in vitro and in vivo by two related mechanisms: (1) AF and ADM would effectively kill CSCs and the bulk of regular cancer cells, respectively. (2) The presence of AF would increase intracellular accumulation of ADM, and thus enhance its therapeutic activity.

It has been known previously that the key targets of AF are TrxR1 and TrxR2, the enzymes which are essential for maintaining cellular redox homeostasis^19,34,35^, and are over-expressed in a variety of cancer cells^36–38^. Inhibition of TrxR alters cellular redox status and leads to accumulation of cellular ROS, causing cellular oxidative stress and cell death when ROS stress is severe. AF has been demonstrated to have potent anticancer effect in a variety of cancers^39^. The therapeutic efficacy of AF against cancer as well as its relative safety profile in patients suggests that this compound could be an attractive drug for clinical treatment of cancer^40,41^. Indeed, AF is currently in phase I/II clinical trials for the treatment of advanced or recurrent NSCLC and small cell lung cancer (http://clinicaltrials.gov/ct2/show/NCT01737502).

It is known that cancer stem cells have low ROS when compared to the non-stem cell counterparts^42^. One possible reason for CSCs to maintain low ROS level could be that a low level of ROS would minimize oxidative damage to genomic materials of CSCs to preserve stemness. In our study, AF treatment of A549 or NCI-H460 cells led to ROS increase, consistent with inhibition of TrxRs and disruption of their role in ROS metabolism. Thus, induction of ROS stress is one of the mechanisms by which AF causes depletion of SP cells. It may be also possible that the AF-induced ROS increase triggers differentiation of SP cells rather than killing them, which might be a reason why SP sub-population of the AF-treated A549 cells recovered to the normal level gradually after the removal of AF.

One major challenge in cancer treatment is drug resistance due to the existence of CSCs^6^. High intracellular ATP levels are associated with drug resistance and stemness^43^. Other factors that contribute to drug resistance include increased drug efflux, enhanced DNA repair capacity, fast drug inactivation, and overexpression of anti-apoptotic proteins^44^. All these abnormal phenomena are associated with ATP consumption. Zhou et al.^43^ reported that intracellular ATP level was a key determinant of chemoresistance in colon cancer cells. Delivery of exogenous ATP into drug-sensitive cells is sufficient to induce drug resistance. In contrast, depletion of intracellular ATP by glycolytic inhibition could enhance the sensitivity of drug-resistant cells to multiple chemotherapeutic drugs. Our previous study has confirmed that the stem-like SP cells have higher cellular ATP content compared with non-SP cells. In the current study, we showed that depletion of cellular ATP by AF could preferentially kill stem-like SP cells due in part to inhibition of the glycolytic enzyme HK. This inhibition of energy metabolism, together with the ability of AF to promote ROS generation, may explain why this compound was highly effective in elimination of stem-like cancer cells. Further studies of AF and its combination with conventional chemotherapeutic agents in clinically relevant setting are needed to evaluate the potential therapeutic applications. In conclusion, our results for the first time show that AF eliminates stem-like cancer cells by ROS increase and glycolytic inhibition. The combination of AF with chemotherapeutic drug adriamycin demonstrates a synergistic effect both in vitro and in vivo, demonstrating that a combination of AF with conventional anticancer drugs would provide a promising new strategy to enhance therapeutic activity.

## Materials and methods

### Chemicals and reagents

Auranofin was purchased from Cayman Chemical Company (Ann Arbor, Michigan, USA). This compound was dissolved in dimethyl sulfoxide (DMSO) as a stock solution of 10 mM and stored in aliquots at −80 °C. ADM was obtained from Shenzhen Main Luck Pharmaceuticals Inc. (Shenzhen, China). CM-H_2_DCF-DA, Hoechst 33342, and verapamil were purchased from Sigma (St. Louis, MO, USA). Rabbit monoclonal anti-Sox2, rabbit polyclonal anti-ABCG2, rabbit polyclonal anti-Oct4, and mouse monoclonal anti-β-actin were purchased from Cell Signaling Technology (Danvers, MA, USA).

### Cell lines

Human non-small cell lung cancer A549 and NCI-H460 cell lines were obtained from the American Type Culture Collection (Rockville, MD, USA). The cells were cultured in RPMI1640 medium supplemented with 10% fetal bovine serum. Human glioblastoma stem cells GSC11 and GSC23 were cultured in serum-free DMEM/F12 medium as described previously^11^. All cell lines were grown in a humidified chamber at 37 °C with 5% CO_2_.

### Assessment of cellular ROS

A549 cells were seeded in six-well plates at 2 × 10^5^ cells per well overnight, and treated with AF or H_2_O_2_ as indicated. Then the cells were incubated with CM-H_2_DCF-DA (10 µmol/L) at 37 °C for 30 min. Cells were washed with PBS, and cellular ROS contents were measured using a Cytomix FC500 flow cytometer (Beckman Coulter, Fullerton, CA, USA).

### Determination of glycolytical activity

Cells were seeded in triplicate at 1 × 10^6^ cells per well in six-well plates and incubated with 10 mM or 100 mM 2-DG for 24 h. Culture media were removed for analysis of lactate levels by an SBA-40C Biosensor (Biology Institute of the Shandong Academy of Science, Jinan, Shandong Province, China).

### Cytotoxicity assay

Cytotoxicity was measured using MTS assay. Cells were seeded in a 96-well plate (2000 cells per well) and incubated with AF and ADM for 72 h. Overall, 20 µL MTS solution was then added to each well and incubated for another 4 h at 37 °C. The optical density (OD) at 490 nm was determined using a Multiskan plate reader (Thermo Scientific, Helsinki, USA). Cell survival rate was calculated as the proportion of survival cells after a drug treatment relative to the untreated control cells.

### Colony formation assay

Cells were seeded at a low density (400 cells per well) in six-well plates, and cultured at 37 °C for 14 days in medium with or without drugs. At the end of incubation, the cells were fixed with formalin for 15 min, stained with crystal violet for 15 min, and the numbers of colonies were counted using AlphaImager HP system (ProteinSimpre, CA, USA).

### Apoptosis assay

Cell apoptosis was analyzed by an Annexin-V/7-AAD (BD) according to the manufacturer’s protocol. Cells were collected and analyzed on Beckman flow cytometer.

### Western blot analysis

Cells were washed twice with ice-cold PBS and lysed in lysing buffer. The concentration of proteins was measured using BCA protein assay (ThermoFisher, Rockford, IL, USA). Cellular proteins were run on a standard SDS-PAGE and transferred to PVDF membranes. Subsequently, the membranes were blotted with specific primary antibodies overnight at 4 °C. The membranes were then incubated with appropriate horseradish peroxidase-conjugated secondary antibodies, and the signals were revealed by the ECL detection system (Keygen Biotech. Co., Ltd, Nanjing, China). β-actin was blotted as a loading control.

### Stem cell sphere-forming assay

GSC11 and GSC23 cells were seeded in six-well plates at a density of 800 cells per well. Cells were cultured in serum-free DMEM/F12 medium and incubated with the indicated concentration of AF. The numbers of tumor spheres were counted at day 12.

### Analysis of SP cells

The SP analysis was performed using a flow cytometry method as described previously^45^. Briefly, viable cells were washed with PBS and re-suspended in pre-warmed RPMI1640 medium containing 2% FBS at a density of 1 × 10^6^ cells/mL. Cells were pre-incubated with or without verapamil (50 μM), an inhibitor of ABC transporters, for 10 min at 37 °C. Hoechst 33342 dye (5 μg/mL) was then added, and the cells were incubated for 90 min at 37 °C in darkness, with shaking every 15 min. After incubation, cells were washed with ice-cold PBS, and kept on ice for flow cytometric analysis using a MOFloXDP Cell Sorter (Beckman Coulter).

### Measurement of cellular ATP

Cellular ATP concentration was determined using an ATP-based CellTiter-Glo Luminescent Cell Viability Kit (Promega, Madison, WI, USA) following the procedures modified from the manufacturer’s instructions. Briefly, cells were seeded in 96-well plates and cultured overnight to allow attachment. Cells were then treated with AF (4, 8 μmol/L) for 6 h. After drug treatment, the plate was equilibrated at room temperature for 30 min. Equal volume of the CellTiter-Glo reagent was added to each well and rocked for 2 min to induce cell lysis. The samples were kept at room temperature for another 10 min. The ATP contents were recorded as luminescent signal, using a luminescent plate reader (Thermo, Fisher Varioskan Flash, Waltham, MA, USA).

### Assays of cellular glucose uptake and lactate production

A549 cells were seeded in culture plates. After attachment, the culture medium was replaced with fresh medium containing various concentrations of AF. After incubation for 12 h, the culture medium from each sample was collected for analysis of glucose and lactate contents, using a Biosensor Analyzer (Biology Institute of Shandong Academy of Sciences, Shandong, China).

### Assay of intracellular ADM

The cellular accumulation of ADM was detected using flow cytometer (Cytomix FC500, Beckman Coulter, Fullerten, CA, USA). In brief, cells were cultured in six-well plates with exposure to ADM with or without AF as indicated. The cells were then collected, washed and re-suspended in cold PBS. The fluorescence indensity of ADM was determined using a flow cytometer.

### Hexokinase enzymatic activity assay

HK activity was measured using a hexokinase assay kit (ScienCell Research Laboratories, Carlsbad, CA, USA) according to the assay procedures recommended by the manufacturer. Purified hexokinase was pre-incubated with various concentrations of AF for 10 min, and then mixed with the assay substrates to start the reaction. The changing NADPH absorbance was monitored in dark for 60 min, using a Multiskan plate reader (Thermo Scientific). For analysis of HK activity in cell lysates, A549 cells were treated with various concentrations of AF for 6 h, and protein extracts were prepared and immediately used for assay of HK activity using the substrate mixtures provided in the assay kit.

### Animal study

For evaluation of tumorigenicity, A549 cells were treated with 4 µM AF for 24 h in vitro. The viable cells were harvested, washed, and re-suspended in serum-free RPMI1640 medium and inoculated subcutaneously into the flanks of athymic nude mice with 5 × 10^4^ or 1 × 10^5^cells per injection site. The animals were then monitored for tumor formation incidence without further drug treatment.

To test the therapeutic effect of AF, ADM, or their combinations in vivo, 1.5 × 10^6^ A549 cells in 100 µL serum-free medium were injected subcutaneously into the right flank of athymic mice. When tumor xenografts were established and measurable after about 7 days, the tumor-bearing mice were randomly divided into four groups and then received the following treatment: (1) Treatment with PBS as control; (2) Treatment with AF, 10 mg/kg, i.p. every day (five times per week, Monday to Friday) for 6 weeks; (3) Treatment with ADM, 5 mg/kg, i.v., once a week for 6 weeks; (4) Treatment with AF + ADM as described in (2) and (3). After 6 weeks of drug treatment, the animals were observed for additional 2 weeks without treatment before termination of the study. Tumor sizes were measured every other day using a caliper, and the tumor volume was calculated using the following formula: Volume = (Length × Width^2^)/2. Body weight of mice were also measured and recorded. At the end of the experiment, mice were killed, and tumors were collected, photographed, and weighed. The animal study was conducted in compliance with a protocol approved by the Institutional Animals Care and Use Committee of Sun Yat-sen University Cancer Center.

### Statistical analysis

Data were presented as mean ± SD. The statistical significance of differences was determined using the Student’s *t*-test. *p* value < 0.05 was regarded as statistically significant.

## Electronic supplementary material

Supplementary Information

## References

[1] Clarke MF (2006). Cancer stem cells—perspectives on current status and future directions: AACR Workshop on cancer stem cells. Cancer Res..

[2] Crea F, Danesi R, Farrar WL (2009). Cancer stem cell epigenetics and chemoresistance. Epigenomics.

[3] Reya T, Morrison SJ, Clarke MF, Weissman IL (2001). Stem cells, cancer, and cancer stem cells. Nature.

[4] Gasch C, Ffrench B, O’Leary JJ, Gallagher MF (2017). Catching moving targets: cancer stem cell hierarchies, therapy-resistance & considerations for clinical intervention. Mol. Cancer.

[5] Golebiewska A, Brons NH, Bjerkvig R, Niclou SP (2011). Critical appraisal of the side-population assay in stem cell and cancer stem cell research. Cell Stem Cell.

[6] Dean M, Fojo T, Bates S (2005). Tumour stem cells and drug resistance. Nat. Rev. Cancer.

[7] Takebe N (2015). Targeting Notch, Hedgehog, and Wnt pathways in cancer stem cells: clinical update. Nat. Rev. Clin. Oncol..

[8] Plaks V, Kong N, Werb Z (2015). The cancer stem cell niche: how essential is the niche in regulating stemness of tumor cells?. Cell Stem Cell.

[9] Kuhlmann JD, Hein L, Kurth I, Wimberger P, Dubrovska A (2015). Targeting cancer stem cells: promises and challenges. Anticancer Agents Med. Chem..

[10] Liu PP (2014). Metabolic regulation of cancer cell side-population by glucose through activation of the Akt pathway. Cell Death Differ..

[11] Yuan S (2013). Effective elimination of cancer stem cells by a novel drug combination strategy. Stem Cells.

[12] Yan B, Dong L, Neuzil J (2016). Mitochondria: an intriguing target for killing tumour-initiating cells. Mitochondrion.

[13] Yan B (2015). Mitochondrially targeted vitamin E succinate efficiently kills breast tumour-initiating cells in a complex II-dependent manner. BMC Cancer.

[14] Zhou D, Shao L, Spitz DR (2014). Reactive oxygen species in normal and tumor stem cells. Adv. Cancer Res..

[15] Dayem AA, Choi HY, Kim JH, Cho SG (2010). Role of oxidative stress in stem, cancer, and cancer stem cells. Cancers.

[16] Chaffman M, Brogden RN, Heel RC, Speight TM, Avery GS (1984). Auranofin. A preliminary review of its pharmacological properties and therapeutic use in rheumatoid arthritis. Drugs.

[17] Chen X (2014). Anti-rheumatic agent auranofin induced apoptosis in chronic myeloid leukemia cells resistant to imatinib through both Bcr/Abl-dependent and -independent mechanisms. Oncotarget.

[18] Chen X, Shi X, Wang X, Liu J (2014). Novel use of old drug: anti-rheumatic agent auranofin overcomes imatinib-resistance of chronic myeloid leukemia cells. Cancer Cell Microenviron.

[19] Fiskus W (2014). Auranofin induces lethal oxidative and endoplasmic reticulum stress and exerts potent preclinical activity against chronic lymphocytic leukemia. Cancer Res..

[20] Ellis P (2004). SOX2, a persistent marker for multipotential neural stem cells derived from embryonic stem cells, the embryo or the adult. Dev. Neurosci..

[21] Robey RW (2009). ABCG2: a perspective. Adv. Drug Deliv. Rev..

[22] Zaehres H (2005). High-efficiency RNA interference in human embryonic stem cells. Stem Cells.

[23] Yuan S (2015). Metabolic activation of mitochondria in glioma stem cells promotes cancer development through a reactive oxygen species-mediated mechanism. Stem Cell Res. Ther..

[24] Pelicano H, Martin DS, Xu RH, Huang P (2006). Glycolysis inhibition for anticancer treatment. Oncogene.

[25] Gazzano E (2016). Overcoming multidrug resistance *by* targeting mitochondria with NO-donating doxorubicins. Bioorg. Med. Chem..

[26] Gottesman MM (2002). Mechanisms of cancer drug resistance. Annu. Rev. Med..

[27] Al-Hajj M, Wicha MS, Benito-Hernandez A, Morrison SJ, Clarke MF (2003). Prospective identification of tumorigenic breast cancer cells. Proc. Natl Acad. Sci. USA.

[28] Singh SK (2003). Identification of a cancer stem cell in human brain tumors. Cancer Res..

[29] Ho MM, Ng AV, Lam S, Hung JY (2007). Side population in human lung cancer cell lines and tumors is enriched with stem-like cancer cells. Cancer Res..

[30] Li C (2007). Identification of pancreatic cancer stem cells. Cancer Res..

[31] Lapidot T (1994). A cell initiating human acute myeloid leukaemia after transplantation into SCID mice. Nature.

[32] Li Y, Atkinson K, Zhang T (2017). Combination of chemotherapy and cancer stem cell targeting agents: preclinical and clinical studies. Cancer Lett..

[33] Wang J (2017). Inhibition of cancer growth in vitro and in vivo by a novel ROS-modulating agent with ability to eliminate stem-like cancer cells. Cell Death Dis..

[34] Fan C (2014). Enhancement of auranofin-induced lung cancer cell apoptosis by selenocystine, a natural inhibitor of TrxR1 in vitro and in vivo. Cell Death Dis..

[35] Pessetto ZY, Weir SJ, Sethi G, Broward MA, Godwin AK (2013). Drug repurposing for gastrointestinal stromal tumor. Mol. Cancer Ther..

[36] Kim HJ (2003). Preferential elevation of Prx I and Trx expression in lung cancer cells following hypoxia and in human lung cancer tissues. Cell Biol. Toxicol..

[37] Lincoln DT, Ali Emadi EM, Tonissen KF, Clarke FM (2003). The thioredoxin-thioredoxin reductase system: overexpression in human cancer. Anticancer Res..

[38] Raffel J (2003). Increased expression of thioredoxin-1 in human colorectal cancer is associated with decreased patient survival. J. Lab. Clin. Med..

[39] Mirabelli CK (1985). Evaluation of the in vivo antitumor activity and in vitro cytotoxic properties of auranofin, a coordinated gold compound, in murine tumor models. Cancer Res..

[40] Glennas A (1997). Auranofin is safe and superior to placebo in elderly-onset rheumatoid arthritis. Br. J. Rheumatol..

[41] Roder C, Thomson MJ (2015). Auranofin: repurposing an old drug for a golden new age. Drugs Ramp. D.

[42] Diehn M (2009). Association of reactive oxygen species levels and radioresistance in cancer stem cells. Nature.

[43] Zhou Y (2012). Intracellular ATP levels are a pivotal determinant of chemoresistance in colon cancer cells. Cancer Res..

[44] Longley DB, Johnston PG (2005). Molecular mechanisms of drug resistance. J. Pathol..

[45] Goodell MA, Brose K, Paradis G, Conner AS, Mulligan RC (1996). Isolation and functional properties of murine hematopoietic stem cells that are replicating in vivo. J. Exp. Med..

