# Supplementary material for: Elimination of stem-like cancer cell side-population by auranofin through modulation of ROS and glycolysis
Source: Cell Death Dis. 2018 Jan 24;9(2):89. doi: 10.1038/s41419-017-0159-4 (PMC5833411; doi:10.1038/s41419-017-0159-4)
Supplement: Supplementary file 1 — Supplementary Information [file 41419_2017_159_MOESM1_ESM.pdf]

Supplementary Information

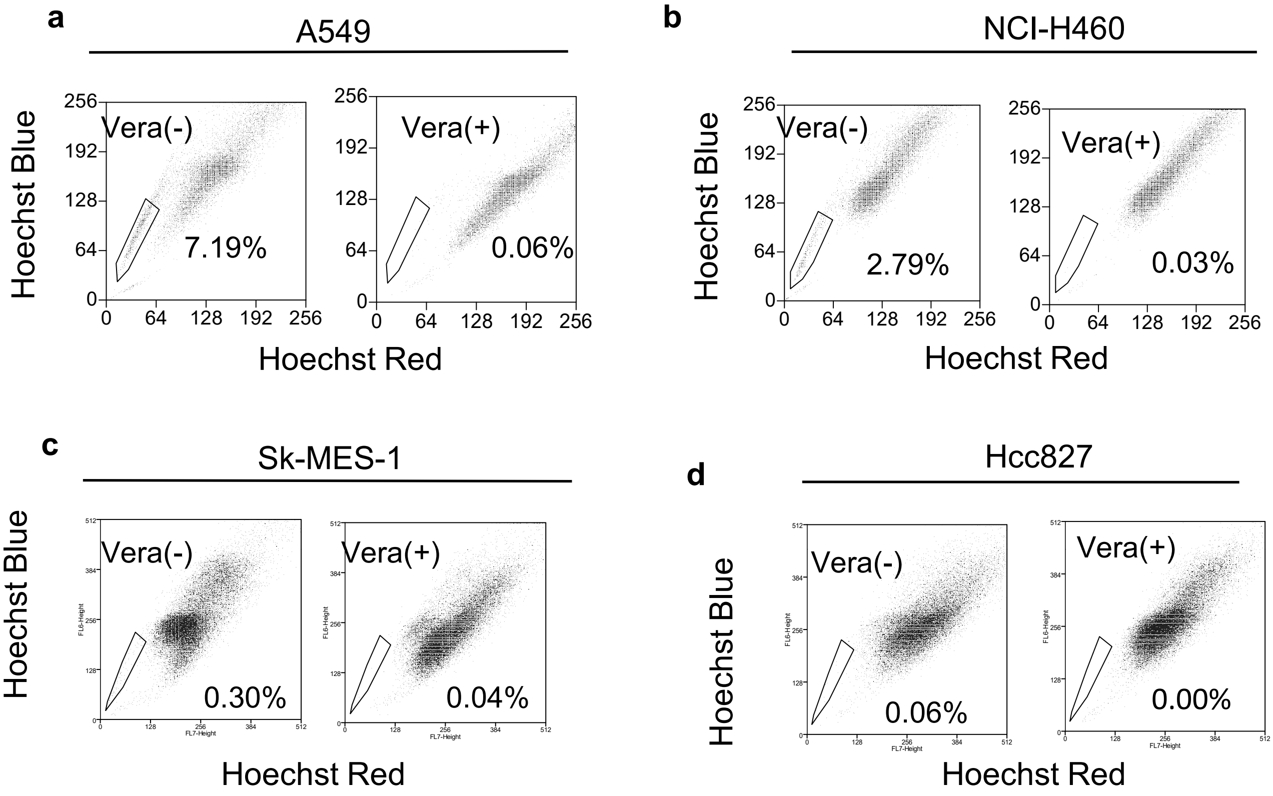

Supplementary Figure 1. Percentage of SP cells in four lung cell lines. (**a-d**) We detected SP cells of four lung cell lines A549 (a), NCI-H460 (b), Sk-MES-1 (c), and Hcc827 (d). Cells were collected and analyzed for SP cells by flow cytometry. The number within each panel shows % of SP cells in the total cell population.

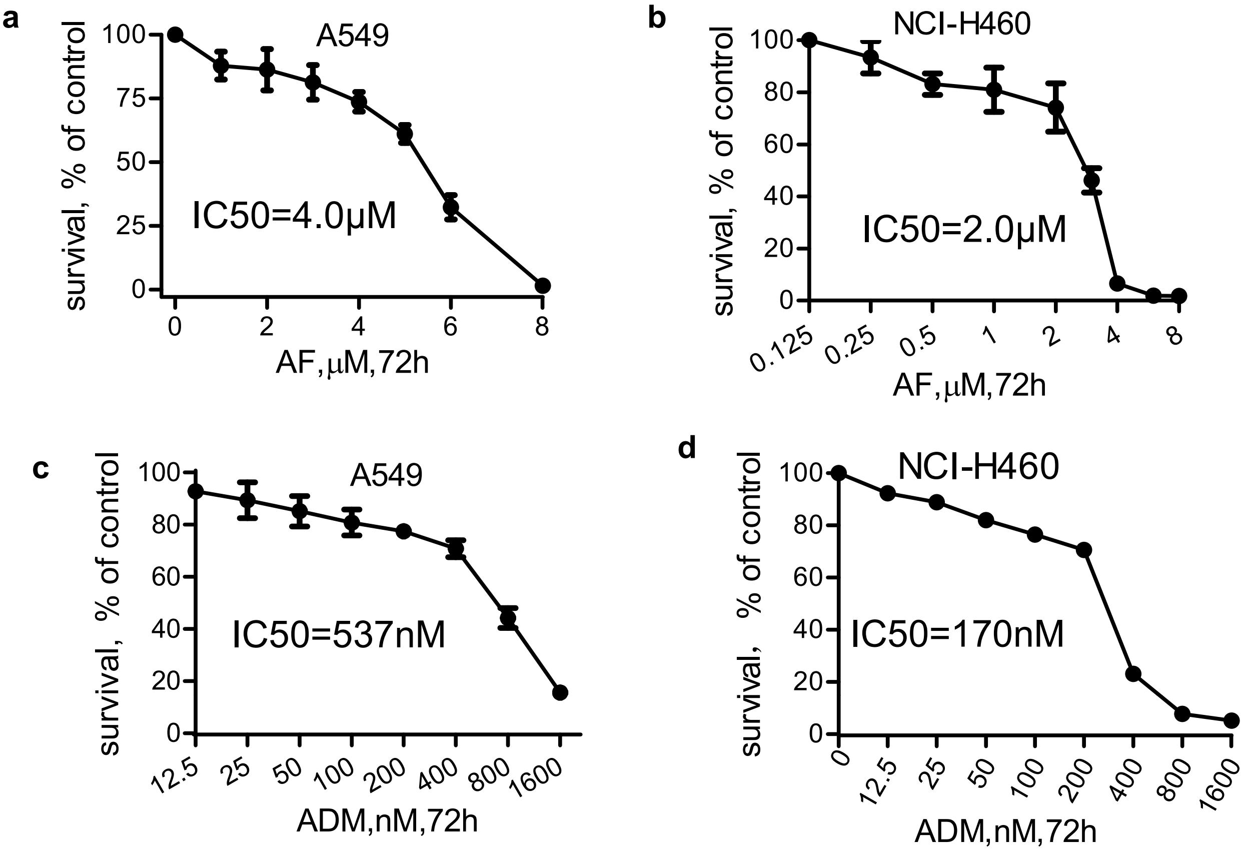

Supplementary Figure 2. Effect of auranofin (AF) and adriamycin (ADM) on cell viability of A549 and NCI-H460 cells. (**a-d**) Cells were seeded in a 96-well plate at the density of 2000/well, and treated with various concentrations of drugs as indicated. After 72 hours, 20 μL MTS solution was added to each well and incubated for another 4 hours. The optical density (OD) was measured at 490 nm using a Multiskan plate reader. Each bar represents mean ± SD, n=3 separate experiments.

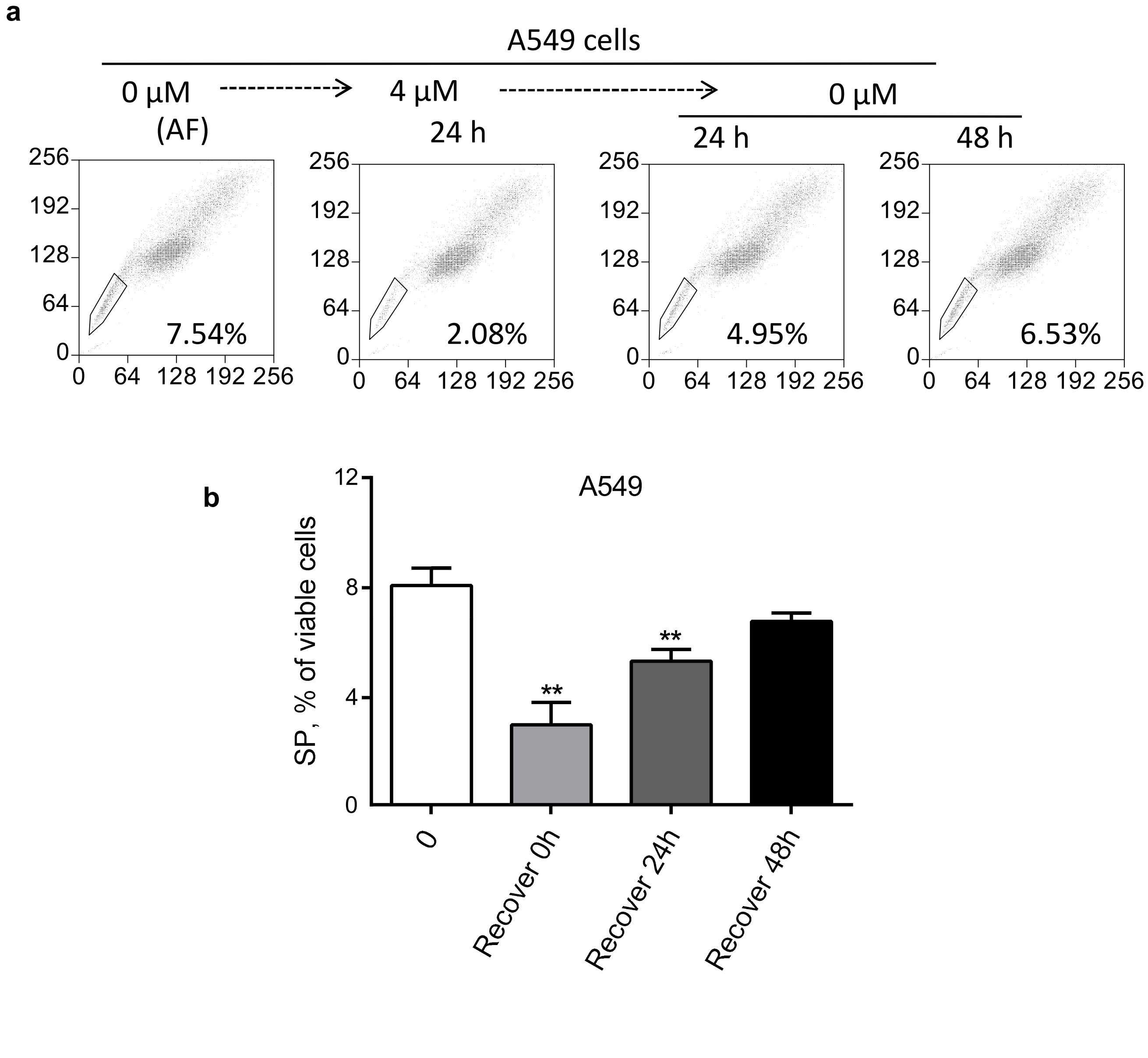

Supplementary Figure 3. Reversible depletion of SP cells by AF. (a) A549 cells were incubated with 4 μM AF for 24 hours, and then switched to fresh medium without AF for another 24 or 48 hours. The cells were collected at each time point and analyzed for SP cells by flow cytometry. The number within each panel shows % of SP cells in the total cell population. **(b)** Quantitative data (mean +/- SD) of three separate experiments as described in **(a).**

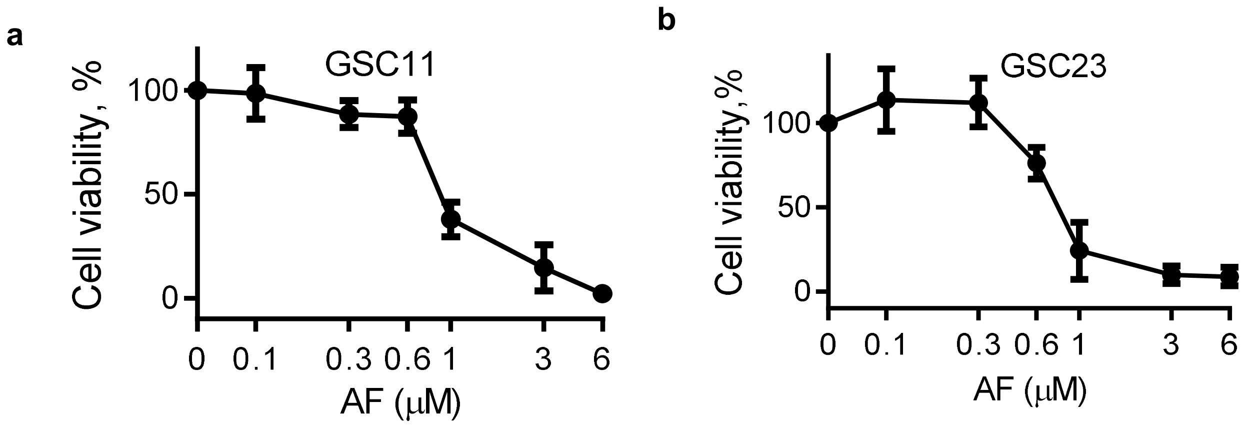

Supplementary Figure 4. Effect of auranofin (AF) on cell viability of GSC cells. **(a,b)** Human glioma stem cell lines GSC11and GSC23 were incubated with the indicated concentrations of AF for 72 hours, and cell viability was measured by MTS assay. Each bar represents mean ± SD, n=3 separate experiments.

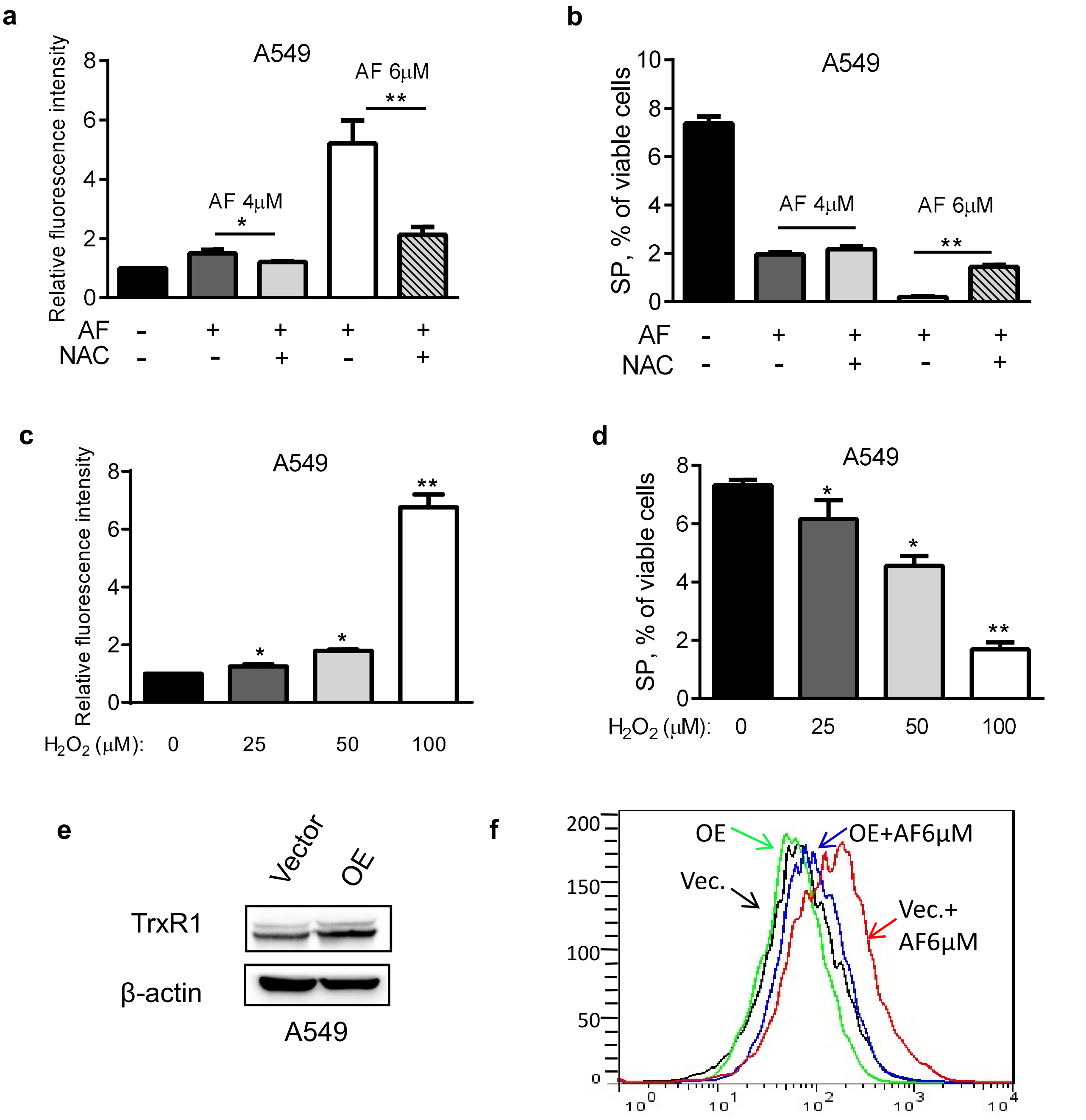

Supplementary Figure 5. Role of ROS in mediating auranofin-induced depletion of SP cells. (a-d) Quantitative data of three separate experiments described in **Figure 3.** Each bar represents mean ± SD, , n=3 separate experiments ,*, p<0.05; **, p<0.01. (e) Western blot analysis of A549 transfected with lentivirus carrying no (A549-Vector, Vec.) or TrxR1 cDNA (A549-OverExpression, OE). (f) ROS level after AF(6μM) treatment in A549-Vector and A549-OverExpression (6h).

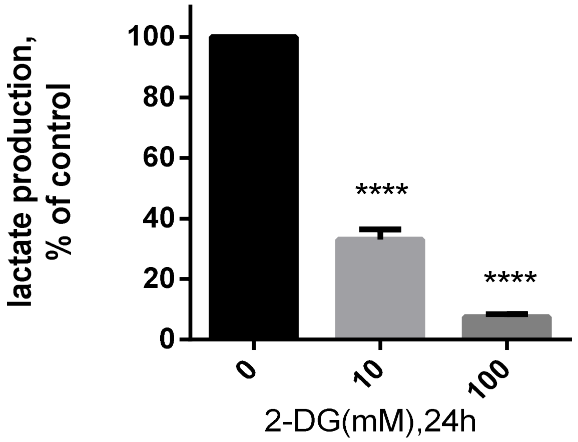

Supplementary Figure 6. Effect of 2-DG on lactate production of A549 cells.

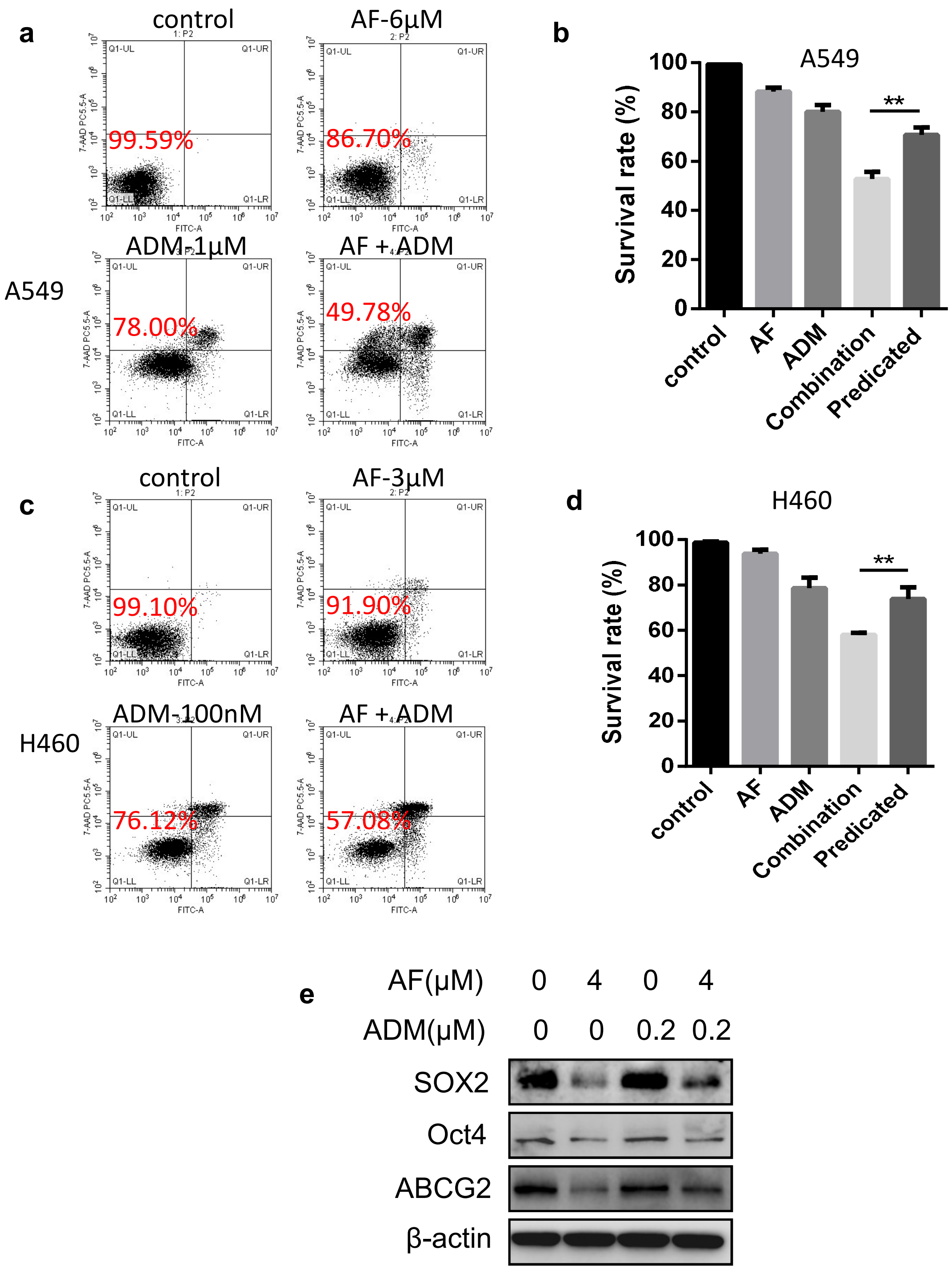

Supplementary Figure 7. Synergistic activity of auranofin (AF) in combination with adriamycin (ADM) on lung cancer cells. (a,c) A549 and NCI-H460 cells were incubated with AF(6 or 3 μM) or ADM(1 or 0.1 μM) or their combinaton for 48h. Apoptosis was determined by flow cytometry. Numbers indicated viable cell percentage. **(b,d)** Comparison of the combination (AF+ADM) effect on apoptosis with the predicted additive effect calculated based on the effect of each drug. **(e)** A549 was treated with AF(4 μM) and ADM(0.2 μM) for 24h. Western blot showed that AF and the combination could reduce the stem cell marker expression. Each bar represents mean ± SD of three separate measurements, **, $p<0.01$.
